# On cosmology in the laboratory

**DOI:** 10.1098/rsta.2014.0354

**Published:** 2015-08-28

**Authors:** Ulf Leonhardt

**Affiliations:** Weizmann Institute of Science, Rehovot 76100, Israel

**Keywords:** transformation optics, analogues of gravity, black holes

## Abstract

In transformation optics, ideas from general relativity have been put to practical use for engineering problems. This article asks the question how this debt can be repaid. In discussing a series of recent laboratory experiments, it shows how insights from wave phenomena shed light on the quantum physics of the event horizon.

## 1. Introduction

Transformation optics [1–7] has been inspired by concepts taken from general relativity [4], in particular by the equivalence of optical media and space–time geometries [8]. What can general relativity learn from transformation optics? In this article, I briefly discuss an example where cosmology can indeed benefit from experience gained with optical media: the quantum physics of black holes [9–12]. This subject is related to experiments with analogies of the event horizon [13–23].

At the event horizon of the black hole, three different areas of physics come together: general relativity, quantum mechanics and thermodynamics. Bekenstein [9] realized that horizons are thermodynamic objects, with the horizon area being proportional to the entropy. Hawking [10] predicted that horizons emit thermal radiation due to quantum physics and calculated the effective temperature of the black hole:
1.1
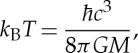
where *M* is the mass of the black hole, 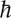 Planck's constant divided by 2*π*, *G* Newton's constant, *c* the speed of light in vacuum and *k*_B_ Boltzmann's constant. Unruh [11] simplified Hawking's argument by showing that an accelerated observer perceives the quantum vacuum as thermal radiation with a temperature that is proportional to the acceleration. These radiation phenomena are part of a wider class of quantum effects [12] based on the physics of horizons. The natural constants in Hawking's formula (1.1) hint at a hidden connection between general relativity, quantum mechanics and thermodynamics that someday may manifest itself in a comprehensive theory. Indeed, Jacobson [24] showed that Einstein's equations follow from postulating the entropy as the area of causal horizons: general relativity is deduced from a thermodynamic equation of state. Discovering the statistical mechanics that underlies the thermodynamics of horizons would probably amount to unifying quantum mechanics with general relativity, one of the greatest challenges of theoretical physics.

Yet, there is a problem with the quantum physics of horizons known as the *trans-Planckian problem* [25]. At the event horizon time stands still and so waves oscillate with wavelengths that become infinitely short. The quanta emitted in Hawking radiation must therefore originate from waves that probe distances beyond all scales—certainly beyond the Planck scale where one might reasonably doubt that the laws of physics are known. Bekenstein's entropy [9], the macroscopic quantity one wishes to use as the benchmark for microscopic quantum theories of gravity, may depend on microscopic physics itself. Unfortunately, one cannot simply test Bekenstein's and Hawking's theory by observing Hawking radiation in astrophysics, for the following reasons [26]. Black holes created from collapsed stars have a minimal mass of about 1.39 solar masses given by the Chandrasekhar limit. The more massive black holes are, the less is their Hawking temperature, as we see from Hawking's formula (1.1). At the minimal mass, the Hawking temperature is nearly eight orders of magnitude below the temperature of the cosmic microwave background. The odds of distinguishing the faint glow of Hawking radiation from the astronomical background are thus truly astronomical.

However, studying the role of trans-Planckian physics is exactly the place where insights from laboratory analogues of gravity will become useful: in an analogous system the microscopic physics is known and one can find out empirically how strong its influence on Hawking radiation is. Extrapolating from laboratory experiments, one may get a level of confidence in astrophysical predictions that is hard to get otherwise [27].

Analogues of the event horizon are based on a simple, intuitive idea first proposed by Volovik and Unruh. Imagine a moving medium, say a river rushing towards a waterfall. The river gets faster and faster, but the speed of waves the river carries is finite. At one point, the river exceeds the wave speed; this point marks the analogue of the event horizon. Mathematically, one shows that the propagation equation of scalar waves in the space–time geometry of the black hole in suitable coordinates, Painlevé–Gullstrand coordinates [28], is equivalent to the wave equation in moving fluids [29] (see appendix A). At the horizon, the space–time river exceeds the speed of light, the analogous flow *u* exceeds the speed of waves *c*. The Hawking temperature depends on the velocity gradient at the horizon [26,29]:
1.2
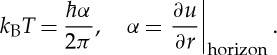


However, it matters which speed the medium matches at the horizon, the group or the phase velocity. Owing to dispersion—the wavenumber dependence of the wave velocity—the group velocity deviates from the phase velocity. The phase velocity *c* is given by *ω*/*k*, the group velocity *v* by ∂*ω*/∂*k*; for *ω*=*c*(*k*)*k* in terms of the wavenumber *k*, the two characteristic velocities agree only for *c*=const., which is never completely the case for waves in media, especially not at horizons, where *k* drastically changes. The role of trans-Planckian physics is played by the dispersion of waves in media. The Planck scale corresponds to the characteristic wavelength over which *c* changes. For sound waves in solids, this scale is the interatomic distance; for sound in Bose–Einstein condensates, it is the healing length [30]. Note that not only does the scale of the dispersion depend on microscopic physics, but also different systems may lead to qualitatively different dispersion relations. Sound waves in solids show normal dispersion, where the phase velocity *c* decreases with increasing wavenumber *k*; sound waves in Bose–Einstein condensates have anomalous dispersion [30], where *c* increases with increasing *k*. One could engineer various types and scales of dispersion and test their influence on Hawking radiation.

So far, experiments in three areas of physics have been performed to test the physics of the event horizon: in optics [13,15,19,20,23], fluid mechanics [14,17,18,22] and the physics of ultracold atoms [16,21]. None of these experiments has unambiguously shown Hawking radiation so far, but most of the bits and pieces required have come together. The paper [18] beautifully illustrates the general idea of analogues of the event horizon with brilliant photographs of the fluid-mechanical phenomenon of the hydraulic jump that occurs when a flowing liquid traverses the speed of waves.

The paper [13] published the idea of realistic tests of Hawking radiation in optics and a simple demonstration of the dramatic frequency shift at horizons that underlies analogues of trans-Planckian physics. The key idea was to create an effective medium that is able to exceed the speed of light in the medium by using light itself. An intense light pulse acts as a moving medium for probe light or, similarly, for the fluctuations of the quantum vacuum. In a subsequent experiment [15], this idea was taken up and applied to the observation of a characteristic radiation at the horizon—in this case the phase-velocity horizon where the speed of the effective medium matches the phase velocity of light. However, the observed radiation exceeded the theoretical prediction by several orders of magnitude [31]. Note that when a medium reaches the phase velocity the frequency becomes zero (for optical analogues the relevant frequency is the Doppler-shifted frequency *ω*′ in the co-moving frame). The problem is that the energy 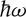 becomes zero as well, and so any small perturbation may be amplified and turn into radiation. This was probably the cause of the radiation seen in the experiment [15]; however further tests need to be done.

In moving fluids, stationary wavy patterns spontaneously appear when the phase velocity *c* matches the flow speed. This phenomenon, called undulation, is familiar to everyone who has been watching streams of water on a rainy day. There one can often see standing ripples appearing on the flowing water. These are not standing waves—they are not waves at all, not oscillations in space and time, but only oscillatory patterns in space: they have zero frequency.

In an early fluid-mechanical experiment [14] and later also in optics [19], a key ingredient of Hawking radiation was seen: the partial conversion of waves with positive wavenumber into waves with negative wavenumber. Waves with negative *k* correspond to the waves behind the horizon of the black hole and they play a crucial role in the creation of quantum particles from vacuum fluctuations at the horizon. The argument is the following. Waves in moving media are subjected to two conservation laws, the conservation of energy and the conservation of norm [26]. The norm of the wave quantifies the amount of particles it carries according to the wave–particle dualism. In moving media, a wave propagating in the positive direction with a positive wavenumber has a positive norm and hence represents a positive amount of particles; a wave with negative wavenumber—but propagating in the positive direction—has a negative norm.^1^ If part of this wave is converted into a negative-wavenumber component, this part would reduce the norm unless the incident wave gets stronger according to the missing norm. Therefore, in conversion processes between waves with positive and negative wavenumbers, waves get amplified. The quantum noise of the amplification is Hawking radiation, the noise temperature is the Hawking temperature (1.1).

One can also use nonlinear optics to create situations where light moves like a fluid and then establish the optical analogue of acoustical horizons. The diffraction of monochromatic light in space appears, in the paraxial approximation, like the evolution of a Schrödinger wave packet in time. Add an optical nonlinearity and the light resembles an interacting quantum fluid, similar to a Bose–Einstein condensate. The optical condensate supports sound waves on a moving background that may very well exceed the speed of sound and hence create acoustic horizons. This idea was demonstrated [20] with a simple thermal nonlinearity, where the absorption of light in a medium changes the refractive index by thermal expansion. However, one may doubt whether such crude experiments with thermal nonlinearities can demonstrate Hawking radiation. Better chances seem to have microcavity polaritons. Cavity polarities are quasi-particles of half-light, half-matter made in a semiconductor waveguide. If the waveguide is strongly confining, the polaritons are interacting like a fluid. This optical fluid is moving and it may carry sound waves. In a recent experiment [23], a supersonic polariton flow was demonstrated that has established a sonic horizon.

In an experiment by Weinfurtner *et al*. [17], stimulated Hawking radiation was observed with waves on flowing water. One could not possibly hope to observe spontaneous Hawking radiation here, i.e. Hawking radiation generated by vacuum fluctuations. The reason is not that water is not a quantum fluid, but rather that the effective Hawking temperatures are below freezing by more than 13 orders of magnitude [17]. However, an incident wave experiences an amplification that one can interpret as stimulated Hawking radiation. The measured amplification gain corresponds to an effective Hawking temperature that was shown to be constant over a broad frequency range (between 0.02 and 0.67 Hz), which indicates that for such experiments Hawking's prediction [10] of a universal radiation temperature is robust. The analogue of trans-Planckian physics in waves on flowing water does not destroy Hawking radiation. However, it is not clear whether the quantitative value of the measured Hawking temperature fits the analogue (1.2) of Hawking's prediction [22].

Atomic Bose–Einstein condensates [30] are quantum fluids that can be sufficiently cold for Hawking radiation to be observable, although it is rather difficult to detect individual quantum particles of sound, phonons, in Bose–Einstein condensates. Here two important phenomena related to Hawking radiation were shown: a flow exceeding the speed of sound without destroying the condensate [16] and the creation of radiation at zero wavenumber [21]. The latter occurred in a so-called black-hole laser [32] that uses two horizons, a white-hole and a black-hole horizon (the white-hole horizon is the time reverse of the black-hole horizon). The two horizons act as both the mirrors and the amplifying medium for Hawking radiation trapped inside. However, the dominating part of the radiation belongs to a wave of zero wavenumber, an undulation, that can be excited by any perturbation and the condensate used was highly non-stationary. It remains to be seen whether the observed wave patterns are indeed created by vacuum fluctuations.

What are the main experimental challenges in the near future? First, it is necessary to gain a better understanding of the effects already seen, of the role of undulations in optics, the black-hole laser and stimulated Hawking radiation with water waves. Second, the most important experiment is still not done: the observation of the quantum signature of Hawking radiation. For this, probably the most feasible method is the measurement of correlated photon pairs in optical experiments [13]. Optics offers several advantages. One is conceptual—light is the simplest quantum system and, unlike waves in fluids, in optics the medium and the wave are clearly distinct from each other. The second advantage is practical—light quanta, photons, are easily detectable. It is perfectly possible that Hawking radiation can be observed in the near future.

The lessons learned from the experiments performed so far [13–23] are threefold: (i) Hawking radiation is a much more general phenomenon than originally anticipated; it belongs to a wide class of wave processes in moving media. (ii) Experiments are surprising: they indicate new aspects of the theory not anticipated in astrophysics, such as the role of undulations or the possibility of black-hole lasing. We have also noted [33–35] that the standard techniques for calculating Hawking radiation need to be changed when confronted with real laboratory systems. (iii) Experiments do not follow theory—theory has to follow experiments. This, presumably, is also true in theoretical astrophysics where, if direct astrophysical observations are impossible, one can still learn for cosmology from the laboratory.

## Footnotes

1 Note that the norm is conserved in the ordinary reflection of waves: upon reflection, both the propagation direction and the wavenumber are changed; hence the norm remains the same.

## Appendix A

In this appendix, I deduce the equivalence between the space–time geometry of the black hole and a moving fluid. Consider the line element of the black-hole geometry in Painlevé–Gullstrand coordinates [28]:

A 1

where *t* and *r* are the Painlevé–Gullstrand coordinate time and radius, respectively, and d*Ω*^2^ the spherical line element. The function *β* depends on *r* as

A 2

where *r*_s_ denotes the Schwarzschild radius

A 3

In order to see the analogy between the Schwarzschild geometry and moving media, we simply express the line element (A 1) as

A 4

with

A 5

and

A 6

Line element (A 4) corresponds to the space–time geometry of waves travelling with velocity *c*. It describes these waves in locally co-moving frames with spatial line element (A 5) that move at the flow speed (A 6). Expression (A 4) thus shows that the Schwarzschild geometry (A 1) corresponds to a moving fluid with velocity profile (A 6). At the horizon, where *r*=*r*_s_, the speed of the space–time flow reaches *c*. According to equation (1.2), the velocity gradient at the horizon determines the Hawking temperature. We obtain from the profile (A 6)

A 7

from which, via equation (1.2), follows Hawking's formula (1.1). Note that the transformation to the co-moving frames is non-relativistic. The black-hole geometry thus corresponds to an effective (non-relativistic!) flow of velocity (A 6). The singularity of the black hole appears like a drain in which the flow disappears. Note that the flow is proportional to  and not to the 1/*r* of an incompressible fluid going down a drain: the effective space–time flow is compressible. Apart from these minor physical imperfections, the analogy between light propagation at the black hole and wave propagation in a moving fluid is mathematically perfect.
